# Embolization of Renal Cell Carcinoma Skeletal Metastases Preceding Orthopedic Surgery

**DOI:** 10.7759/cureus.37961

**Published:** 2023-04-21

**Authors:** Adam A Amado, Hussein Chahrour, Hussam Hindi, Priya Sankaran, Ali Harb

**Affiliations:** 1 Interventional Radiology, Detroit Medical Center (DMC), Detroit, USA

**Keywords:** pre-operative embolization, pathological femoral shaft fracture, hyper vascular metastases, postoperative blood loss, renal cell carcinoma

## Abstract

Renal cell carcinoma (RCC) is the most common type of renal malignancy in adults. Bone is a major site of metastatic disease from RCC. Osseous metastatic disease from RCC is often seen in the spine, pelvis, and femur, and is usually hypervascular in nature like its primary tumor source. This can cause significant pain, reduced function, pathological fracture, nerve compression, and decreased quality of life during cancer treatment and disease course. Surgical treatments for pathological fracture of the femur include resection, reconstruction, and stabilization with arthroplasty or placement of an intramedullary nail. This series looks at three cases of renal cell carcinoma metastases to the hip with pre-procedural embolization and orthopedic stabilization. Interventional radiology embolization of the arterial supply to the metastatic hypervascular bone lesions can reduce intraoperative blood loss and associated complications.

## Introduction

Metastatic disease to the bone can have a painful presentation with associated impending or true pathologic fractures that negatively affect function and quality of life in cancer patients [1]. Treatment of hypervascular bone metastases with internal fixation is associated with a high risk of bleeding, but can improve function and provide pain relief. This is especially relevant in the treatment of renal cell carcinoma (RCC), as the majority of osseous metastases from RCC are hypervascular [2]. Complications associated with the treatment of osseous metastatic disease with open and minimally invasive orthopedic surgical approaches include significant perioperative blood loss requiring blood transfusion, longer procedure times, and intraoperative mortality. Preoperative embolization has been utilized to reduce blood flow to metastatic lesions prior to surgery. Multiple studies have shown a variable reduction in intraoperative blood loss with preoperative embolization of bone metastases. 

## Case presentation

Three cases of embolization of four RCC femoral bone metastases were reviewed. All interventions were followed by orthopedic surgery to stabilize the femur in the setting of pathological fracture or diffuse osteolytic lesions and impending fracture.

Case 1

**Figure 1:**
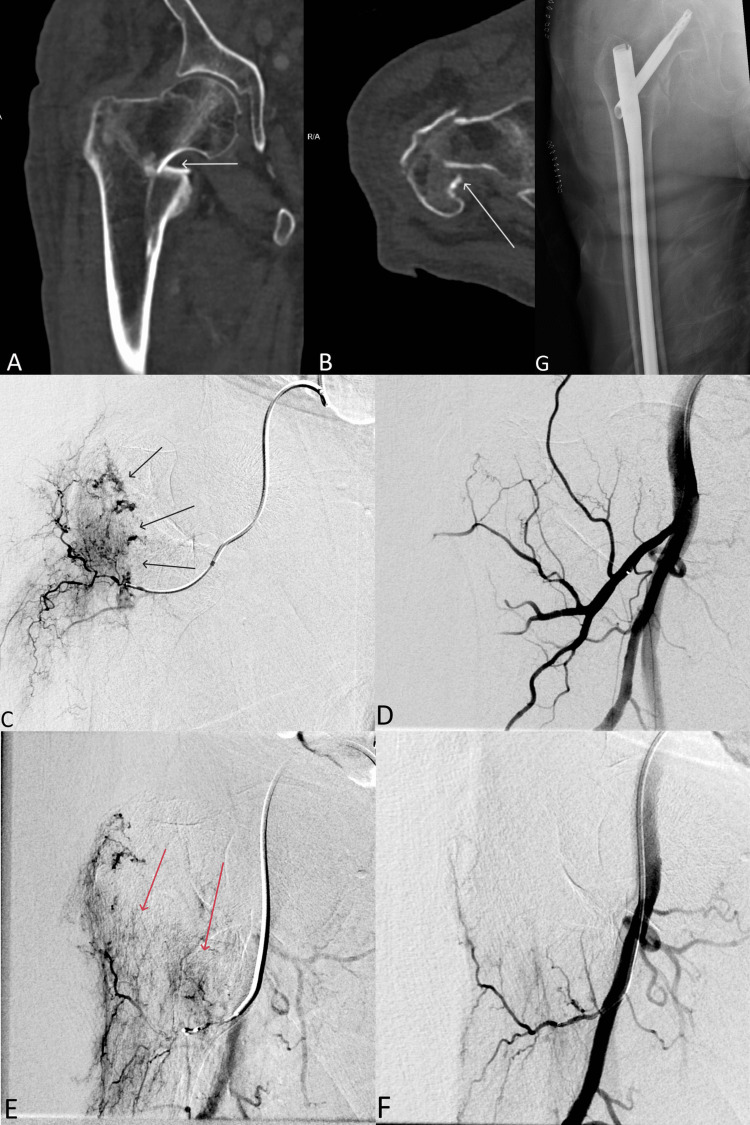
A 78-year-old male with right hip pain. A) B) Coronal and Axial CT of the hip demonstrating an intertrochanteric fracture (white arrows). C) E) Digital subtraction angiography (DSA) demonstrating the areas of tumor blush supplied by the right lateral circumflex (black arrows) and perforating femoral (red arrows) arteries. D) F) DSA demonstrating embolization results. G) Pain film following orthopedic surgery.

Patient is a 78-year-old male with a history of metastatic RCC, presenting with inability to ambulate and right hip pain after a fall. Physical exam demonstrated an externally rotated right lower extremity with pain to palpation. Computed tomography revealed a comminuted, impacted, intertrochanteric fracture of the right femur, with minimal displacement of the greater trochanter, and fragments along the lesser trochanter, with a small hematoma, concerning for a pathologic fracture (Figure 1A, 1B). Interventional radiology (IR) was consulted for preoperative embolization of the suspected metastatic disease, which was performed on the same day. Left femoral artery access was achieved and an angled glide-catheter was advanced over Glidewire (Terumo, Tokyo, Japan) into the right external iliac artery. Tumor blush was seen along the fracture site in the proximal right femur, and two main supply branches were identified (Figure 1C, 1E). A 2.8 French Progreat microcatheter (Terumo, Somerset, NJ, USA) was advanced into a branch of the right lateral circumflex femoral artery and the first perforator from the profunda femoris, 300-500 μm Embospheres (Merit Medical, South Jordan, UT, USA) were injected [3]. Post-embolization angiogram showed resolution of tumor blush (Figure 1D, 1F). The patient had no acute events overnight after the procedure, with maintained good peripheral perfusion. The following day, the patient underwent intramedullary nailing of the right femur with biopsy (Figure 1G). The patient was discharged home three days later.

Hemoglobin was 9.1 g/dl on admission. Estimated blood loss during the orthopedic procedure was 200 mL. Immediately after the procedure, hemoglobin was 8.8 g/dl. Hemoglobin dropped to 7.9 g/dl on the evening of the procedure. On postoperative day two, hemoglobin dropped to 6.7 g/dl, and the patient required a single unit of packed red blood cell (RBC) transfusion. Hemoglobin increased to 7.7 g/dl and was stable thereafter. 

Case 2

**Figure 2:**
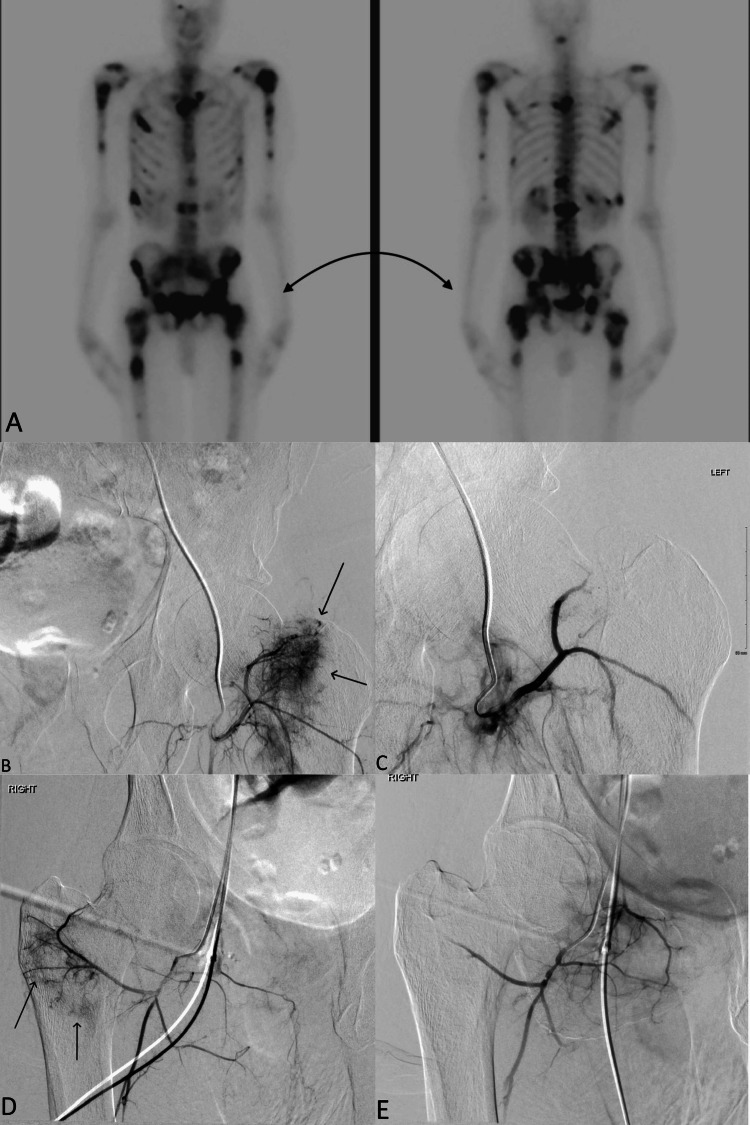
A 49-year-old male with bilateral hip pain. A) Nuclear medicine bone scan demonstrating diffuse osteolytic lesions and extensive involvement of the bilateral femurs (curved arrow). B) D) Digital subtraction angiography (DSA) demonstrating areas of tumor blush supplied by the left and right lateral circumflex femoral arteries (straight arrows). C) E) DSA demonstrating embolization results.

Patient is a 49-year-old male with metastatic RCC. Presenting with worsening lower back and crushing type hip pain bilaterally, associated with weakness and difficulty ambulating. Physical exam revealed reduced lower extremity strength bilaterally to 3/5. Computed tomography demonstrated a primary RCC and multiple metastatic osteolytic lesions, later confirmed with a nuclear bone scan (Figure 2A). Embolization and intramedullary nailing of bilateral femurs were planned as prophylaxis for pathological fractures. Right common femoral artery access was achieved, and a glide-catheter was advanced over Bentson wire (Cook Medical, Bloomington, IN, USA) into the left external iliac artery. Tumor blush was found to be supplied by the left circumflex femoral artery, which was then selected with a 2.8 French Progreat microcatheter. 300-500 μm Embospheres were deployed until stasis of flow was achieved (Figure 2B, 2C). The right external iliac artery was then selected, and tumor blush was found to be supplied by the right circumflex artery. The microcatheter was advanced to target, and 300-500 μm Embospheres were deployed. Successful embolization was demonstrated by resolution of tumor blush (Figure 2D, 2E). Three days later, intramedullary nailing of the bilateral femurs was completed. The patient was discharged one week later without complication. 

Hemoglobin was 10.0 g/dl on admission. Estimated blood loss during the procedure was 650 mL. Hemoglobin decreased to 8.8 g/dl on the day of the orthopedic procedure and subsequently dropped to a low of 6.6 g/dl over the next two days. The patient was transfused with a single unit of packed RBCs, and hemoglobin stabilized at 7.4 g/dl. 

Case 3 

**Figure 3:**
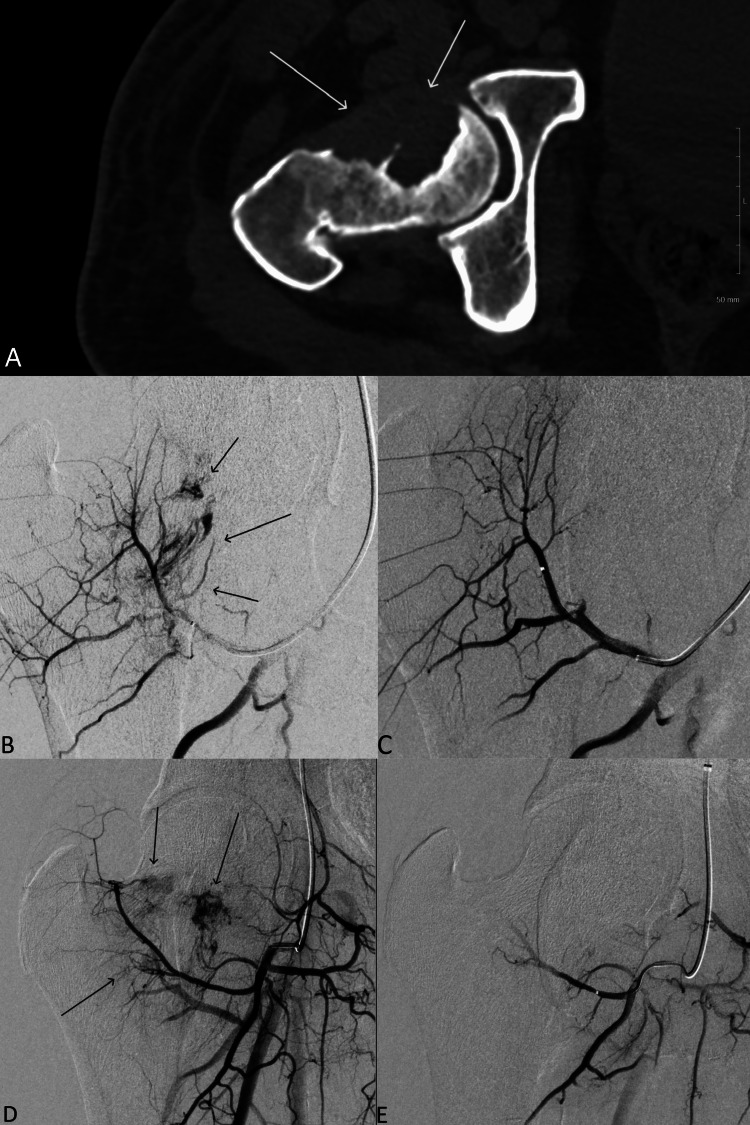
A 52-year-old male with bilateral lower extremity pain and weakness. A) Axial CT of the hip demonstrating lytic destruction of the right femur (white arrow). B) D) Digital subtraction angiography (DSA) demonstrating areas of tumor blush supplied by the branches of the perforating femoral and the left lateral circumflex arteries (black arrows). C) E) DSA demonstrating results of embolization.

Patient is a 52-year-old male presenting with bilateral lower extremity weakness and severe radiating pain for the past six months. Physical exam revealed strength was 3/5 in the lower extremities bilaterally. Computed tomography revealed a large left renal neoplasm, extensive metastases, and lytic destruction of the proximal right femur (Figure 3A). CT-guided biopsy identified the disease as RCC. Prophylactic right hemiarthroplasty with pre-operative IR embolization was planned. The left common femoral artery was accessed and an angled glide catheter was advanced over a Glidewire into the right common iliac artery. Digitally subtracted angiography (DSA) showed a tumor blush supplied by the right lateral circumflex, and a branch of profunda femoris arteries (Figure 3B, 3D). A 2.8 French Progreat microcatheter was advanced into a branch of the profunda femoris. Embolization was performed using 500 to 700 μm Embospheres. The lateral circumflex artery was then selected and embolized with the same. Post-embolization angiography showed resolution of the tumor blush in the right femur (Figure 3C, 3E). The patient was discharged two weeks later, after treatment for multiple acute conditions unrelated to malignancy.

Hemoglobin prior to the orthopedic procedure was 8.5 g/dl. Estimated blood loss during the procedure was 450 mL. Patient received two units of packed RBCs intraoperatively. Over the following three days, hemoglobin fell to a low of 6.1 g/dl before stabilizing at 8 g/dl, after the transfusion of three units of packed RBCs. A hematoma secondary to the orthopedic procedure was identified as the likely source of this acute blood loss. 

## Discussion

It is reported that 30% of RCC patients develop osseous metastatic disease [4]. With the increased prevalence of osseous metastatic disease, orthopedic procedures are providing significant improvement in function and pain relief. These procedures are associated with a high risk of perioperative blood loss due to hypervascularity. Significant intraoperative blood loss increases mortality and requires blood transfusions that have associated risks. Bleeding during the procedure impairs the visualization of bone lesions, and can affect resection techniques and margins [5]. These cases demonstrate the application of pre-operative embolization for orthopedic fixation of true and impending pathological fractures in bone metastases. In all of the cases described, patients did not require massive blood transfusion, and did not have hemodynamic instability requiring pressors or intensive care support.

Due to internal fixation procedures having a high risk of intraoperative blood loss, preoperative embolization should be considered for all patients. Hypervascularity of metastases may be assessed via preoperative imaging, with contrast-enhanced CT or MRI. In our presented cases, we utilized 300-500 μm and 500-700 μm Embospheres to embolize the arterial branches supplying the osseous metastases on a total of four femurs. All cases involved the respective circumflex femoral artery, and two of them (50%) included additional supply from branches of the profunda femoris; subsequent DSA imaging confirmed >95% devascularization. It is suspected that these measures decreased total blood loss. The patients had varying amounts of perioperative blood loss (Average: 325mL, Range: 200-450 mL). Hemoglobin was easily stabilized with transfusions on the medical/surgical floor.

Multiple studies have described a positive effect of pre-operative embolization in patients with RCC bone metastases, with studies reporting an average reduction in blood loss ranging between 290-1000 mL with pre-embolization [1]. Some of the included studies have found that compared to controls, pre-embolization resulted in less blood loss (Embolization: 900±1230 mL. Controls: 1770±2590 mL), less transfusion volume (Embolization: 2.15±3.03 units. Controls: 3.56±5.37 units), and shorter operation time (Embolization:3.13 hours. Controls: 3.91 hours) [6]. Other studies found no difference in transfusion rates between patients who received pre-embolization and those who did not and concluded that pre-embolization should be planned on a case-by-case basis [7]. Smaller studies, that included incomplete devascularization, have not found a statistically significant difference in blood loss between pre-embolized and non-embolized cases [8]. Overall, the reported rate of embolization-related complications is 0-9% [1]. Unfortunately, papers on the subject were based on retrospective cohorts and no randomized control trials were found. In summary, embolization with image-guided, minimally invasive techniques is a low-risk procedure that can be offered to select patients to decrease mortality and improve outcomes in hypervascular bone metastases.

## Conclusions

IR embolization of femoral metastases from RCC can be an effective therapy to reduce blood loss during orthopedic procedures. It can reduce blood supply to bone metastases, and reduce mortality both intra- and post-operatively. All cases of surgical fixation in osseous metastatic disease should be evaluated for possible pre-embolization. The optimal approach for embolization requires further investigation to establish guidelines regarding timing, location of disease, preferred embolic agent, and other factors to reduce operative complications associated with surgical intervention.
